# An *In Vitro* Model of Diabetic Retinal Vascular Endothelial Dysfunction and Neuroretinal Degeneration

**DOI:** 10.1155/2021/9765119

**Published:** 2021-11-10

**Authors:** Qiyun Wang, Xinyuan Zhang, Kaiyue Wang, Ling Zhu, Bingjie Qiu, Xiaosi Chen, Xiao Lin, Yao Nie

**Affiliations:** ^1^Beijing Institute of Ophthalmology, Tongren Eye Center, Beijing Tongren Hospital, Capital Medical Univeristy, Beijing, China; ^2^Beijing Retinal and Choroidal Vascular Diseases Study Group, China; ^3^Save Sight Institute, Department of Ophthalmology, Faculty of Medicine and Health, The University of Sydney, Sydney, Australia

## Abstract

**Background:**

Diabetic retinopathy (DR) is a leading cause of blindness in working-age populations. Proper *in vitro* DR models are crucial for exploring pathophysiology and identifying novel therapeutic targets. This study establishes a rational *in vitro* diabetic retinal neuronal-endothelial dysfunction model and a comprehensive downstream validation system.

**Methods:**

Human retinal vascular endothelial cells (HRMECs) and retinal ganglion cells (RGCs) were treated with different glucose concentrations with mannitol as matched osmotic controls. Cell proliferation and viability were evaluated by the Cell Counting Kit-8. Cell migration was measured using a transwell migration assay. Cell sprouting was assessed by a tube formation assay. The VEGF expression was assessed by ELISA. RGCs were labeled by neurons and RGC markers TUJ1 and BRN3A for quantitative and morphological analysis. Apoptosis was detected using PI/Hoechst staining and TUNEL assay and quantified by ImageJ.

**Results:**

Cell proliferation and migration in HRMECs were significantly higher in the 25 mM glucose-treated group (*p* < 0.001) but lower in the 50 mM and 100 mM groups (*p* < 0.001). The permeability and the apoptotic index in HRMECs were statistically higher in the 25 mM, 50 mM, and 100 mM groups (*p* < 0.05). The tube formation assay found that all the parameters were significantly higher in the 25 mM and 50 mM groups (*p* < 0.001) concomitant with the elevated VEGFA expression in HRMECs (*p* = 0.016). Cell viability was significantly lower in the 50 mM, 100 mM, and 150 mM groups in RGCs (*p*_50mM_ = 0.013, *p*_100mM_ = 0.019, and *p*_150mM_ = 0.002). Apoptosis was significantly elevated, but the proportion of RGCs with neurite extension was significantly lower in the 50 mM, 100 mM, and 150 mM groups (*p*_50mM_ < 0.001, *p*_100*mM*_ < 0.001, and *p*_150mM_ < 0.001).

**Conclusions:**

We have optimized glucose concentrations to model diabetic retinal endothelial (25-50 mM) or neuronal (50-100 mM) dysfunction *in vitro*, which have a wide range of downstream applications.

## 1. Introduction

Diabetic retinopathy (DR) is a leading cause of blindness in the working-age population in both developed and developing countries [1]. DR is the most important neurovascular ocular complications of diabetes mellitus. Identifying new biomarkers for diagnosis and therapeutic targets would greatly benefit patients with DR to prevent vision loss [2].

*In vitro* models of DR have a crucial role in understanding the pathophysiology of the disease and identifying new therapeutic strategies. The effects of high glucose on retinal cell homeostasis and the identification of potentially protective molecules provide deeper insight from *in vitro* studies. We have previously shown that apoptosis of neurons and dysfunctions of the retinal blood barrier are the early and key features of DR. Furthermore, neuronal vascular unit dysfunction was identified as the key pathogenesis of DR [1]. Ganglion cells have been recognized as the earliest damaged retinal neurons under hyperglycemia, proved by our previous studies [3].

There is no universally accepted glucose concentration for *in vitro* studies to simulate human DR. The *in vitro* model of diabetes is currently and commercially established by treating cells with 5.5 mM to 25 mM concentrations of glucose [4–6] and in endothelial cells only. Furthermore, the *in vitro* glucose concentration is not entirely equivalent to the physiological condition of human beings. The monoglucose concentrations are not optimal for simulation of the blood glucose levels in patients with hyperglycemia, especially for DR. Under the external environment, different cell types have different tolerance to high glucose. Nevertheless, the proper concentrations and the effect of the glucose concentration and osmotic pressure on the biological behavior of HRMEC and neurons have not been fully elucidated [7].

In this study, we investigate the influence of the glucose concentrations gradient on the biological characteristics of HRMEC and RGCs [8]. As the osmotic pressure could be a coeffector in a hyperglycemia pathological condition, we also set up serial control groups of gradient increased osmotic pressure to exclude this effect in human endothelial cells.

## 2. Materials and Methods

### 2.1. Cell Culture of Human Retinal Microvascular Endothelial and Retinal Ganglion Cells

Primary HRMECs and RGCs were purchased from Shanghai Xuanya Biotechnology Co., Ltd (Shanghai, China). Dulbecco's modified Eagle's medium (DMEM) powder and fetal bovine serum (FBS) were bought from Gibco Life Technologies (New York, NY, USA). HRMECs were cultured in a 5.5 mM glucose DMEM medium containing 10% FBS in a humidified atmosphere containing 5%CO_2_/95% air at 37°C. Cells passaged for 4 and 8 times were eased for further experiments.

### 2.2. Experimental Grouping

Cells were divided in two groups: the control group, which was cultured in DMEM medium supplemented with 5.5 mM glucose, and the high-glucose group, cultured in DMEM medium supplemented with G25, G50, G100, and G150 (D-glucose concentrations 25, 50, 100, and 150 mmol/L, respectively). The maximum glucose concentration for RGCs is 150 mmol/L. Mannitol was added to the 5.5 mM group to adjust the osmotic pressure to correspond with the same osmotic pressure of G25, G50, G100, and G150, thus forming M25, M50, M100, and M150 (mannitol concentrations 19.5, 44.5, 94.5, and 133.5 mmol/l, respectively). Each experiment was repeated three times.

### 2.3. Evaluation of the Cutoff and Extreme Value of High Glucose in HRMECs and RGCs

A pilot study was designed to evaluate the cutoff and acute value of high glucose for HRMECs and RGCs. A series glucose concentration was set up as follows: 5.5 mM, 10 mM, 15 mM, 25 mM, 35 mM, 50 mM, 75 mM, and 100 mM. Cell proliferative capacity of HRMECs and cell viability of RGCs were assessed via CCK-8 assay before they were treated with different glucose concentrations as previously described [9]. Briefly, cells were seeded in 96-well culture plates (1 × 10^4^cells/well). After 24 h, the cells were mixed with the control or experimental medium and cultured for an additional 24, 48, and 72 h (each treatment group has five repeat wells). At each time point, 10 *μ*l CCK-8 reagent (Dojindo Molecular Technologies, Inc., Kumamoto, Japan) was added to each well and incubated for another 2 h at 37°C. The OD value of each well was detected at the wavelength of 450 nm with a microplate reader (SpectraMax 250; GE Healthcare Life Sciences, Chalfont, UK).

### 2.4. The Endothelial Cell Transwell Migration and Invasion Assay

To investigate the migratory response of endothelial cells to hyperglycemia, HRMECs were incubated in a serum-free-conditioned medium at 37°C with a humidified atmosphere for 24 h before the suspension. 100 *μ*l of serum-free medium (containing 2 × 10^4^ cells) was added to the upper chambers of a transwell plate with the pore of 8 *μ*m (24 wells, Corning, NY, USA), while the lower chambers were added with 600 *μ*l of complete medium with 20% FBS. After incubation for 24 h, 48 h, and 72 h, migrated cells attached to the other side of the insert were fixed with 90% ethanol followed stained with 0.1% crystal violet. Five randomly field (×200) photographs were taken under an inverted microscope (Carl Zeiss, Jena, Germany), and the average number of cells was counted. The experiment was repeated three times.

### 2.5. Detection and Percentage of Apoptotic Cells

Apoptotic cells were detected using a dUTP Nick-End Labeling (TUNEL) kit (#11684795910, Roche, Mannheim, Germany) and PI/Hoechst staining method. TUNEL assay is designed to identify extensive DNA degradation during the late stages of apoptosis *in situ* in the apoptotic cells. Briefly, the cells grown on coverslips were fixed with 4% paraformaldehyde for 1 h at RT. The cells were incubated with permeabilization solution (0.1% Triton X-100 in 0.1% sodium citrate, freshly prepared) for 2 min on ice after washing with PBS. The cells were then incubated with the TUNEL reaction mixture in a humidified chamber in the dark at 37°C for 1 h. After counterstaining with DAPI (1 *μ*g/ml), stained sections were examined by a fluorescence microscope (Carl Zeiss, Jena, Germany). TUNEL-positive nuclei were stained green, and all other nuclei were stained blue.

For PI/Hoechst staining, cells were seeded in 24-well culture plates (2 × 10^4^ cells/well). After each time point of 24 h, 48 h, and 72 h, PI (#M5109, Abmole Bioscience, USA) and Hoechst (#M5113, Abmole Bioscience, USA) were added into each well, and the cells were incubated in the dark for 15 min followed by PBS (5 minutes each) washing for two times. For quantitative analysis, nine fields were randomly photographed under a 200-fold inverted microscope (Carl Zeiss, Jena, Germany) [10]. Apoptosis index was calculated as the number of positive cells (TUNEL or PI-positive nuclei/the number of DAPI or Hoechst-stained cells).

### 2.6. Cell Permeability Assay

To evaluate the effects of hyperglycemia on endothelial cell proliferative ability, cell permeability was tested using the BSA-FITC kit [11, 12]. HRMECs were seeded in the upper Transwell-COL membrane insert (24 wells, Corning Incorporated, NY, USA). After reaching 90% confluence, the HRMECs were starved for 12 h in serum-free medium and subsequently replaced with control or experimental medium for 24 h. After 24 h, a tracer solution of FITC-labeled BSA (250 *μ*g/ml; Sigma-Aldrich, Germany) was added to the upper insert, and 500 *μ*l medium without FITC-BSA was added to the lower insert. After incubation for 2 hours, the collected media were measured using a fluorophotometer (Tecan, Switzerland) at an emission/excitation wavelength of 495/520 nm.

### 2.7. Tube Formation Assay

To evaluate the angiogenic capability under hyperglycemia, 50 *μ*l Matrigel (#356234, BD Biosciences, Oxford, UK) was added to each well of a 96-well plate and incubated for 45 min at 37°C in a humidified atmosphere with 5% CO_2_ as previously described [13, 14]. Until Matrigel was polymerized, 5 × 10^4^ cells in 100 *μ*l DMEM were seeded in each well. Capillary-like tubes were formed within 9 hours and recorded with a video camera (Carl Zeiss, Jena, Germany). Images of tube morphology were obtained at ×100 magnification and quantified by the ImageJ software (NIH Image, Bethesda, MD) [13, 14].

### 2.8. Detection of VEGFA Protein Concentration

To investigate the pathological mechanisms under hyperglycemia, the supernatant of the different groups was harvested and centrifuged at 4°C for 10 min at 3000 × g to remove the cell debris, and then it was stored at -80°C for ELISA. After dilution, the concentration of VEGFA in cell supernatants was quantitatively detected using the Human VEGFA ELISA kit (RayBiotech Systems, USA) following the manufacturer's instruction. Data was quantified in comparison to VEGF standards.

### 2.9. Immunochemistry

RGCs were harvested and seeded in a 24-well culture plate (3 × 10^4^ cells/well). After incubation for 24 h, 48 h, and 72 h, cells were fixed with 4% paraformaldehyde at RT for 30 min according to previously described methods [15–17]. After washing with Hank's Balanced Salt Solution (HBSS basic 1x, Gibco Company, USA), cells were permeabilized in 0.3% Triton (Sigma-Aldrich, Germany) in HBSS for 15 min at RT. The cells were then blocked with a blocking buffer containing 1% bovine serum albumin (BSA) and 5% goat serum in HBSS for 60 minutes after washing with HBSS. After incubation with the primary antibodies, rabbit anti-beta III tubulin (1 : 1000,Cat#ab18207,Abcam,Cambridge, MA), which was specific for neurofilament heavy chains [18], and mouse anti-Brn3a (1 : 25, Cat#sc-8429, Santa, USA), which was specific expression in RGCs nucleus [17, 19] for overnight at 4°C, the cells were washed three times and incubated with secondary antibodies (goat anti-mouse and goat anti-rabbit) (1 : 500, Cat #ab150077, Cat #ab150116, Abcam, Cambridge, MA) at RT for 2 hours with light-shielded and counterstained with DAPI. Five fields were randomly photographed under a 200-fold inverted microscope (Carl Zeiss, Jena, Germany). The number of cells which had neurite lengths equal to or greater than three times the cell body diameter was calculated by ImageJ.

### 2.10. Statistical Analysis

Statistical analysis was performed using the SPSS software version 20.0 (SPSS, Inc., Chicago, IL, USA). Data distribution was assessed by the One-Sample Kolmogorov-Smirnov test. Data were expressed as means ± SD or median (interquartile range). Variance (ANOVA) analysis for continuous variables and chi-square test for noncontinuous variables were applied. The Bonferroni correction was used for multiple-comparison correction. A *p* value less than 0.05 is considered significant.

If the above analysis confirmed any significant difference, a comparison between two groups was then conducted by independent Student's *t*-test for continuous variables and post hoc test to compare the difference between groups for noncontinuous variables. *p* values < 0.05 was considered statistically significant.

## 3. Results

### 3.1. 25 mM and 50 mM May Be Taken as the Cutoff Value of Hyperglycemia for Retinal Vascular Endothelial Cells

Significant difference was found in the optical density (OD) value in the different glucose concentration groups at 24 h, 48 h, and 72 h by the CCK-8 assay (*F*_24h_ = 41.789, *p* < 0.001; *F*_48h_ = 181.054, *p* < 0.001; and *F*_72h_ = 51.189, *p* < 0.001). There was significant difference in the OD value in the groups of 25 mM, 50 mM, and 100 mM compared to the 5.5 mM group (*p* < 0.01), but no difference was found in the 10 mM and 15 mM groups compared to the 5.5 mM control group (*p* > 0.05). The glucose concentration of 25 mM can be regarded as the cutoff value of DR. When the glucose concentration is greater than 50 mM (50 mM, 75 mM, and 100 mM), the proliferation capacity of HREMC was obviously inhibited.

**Figure 1:**
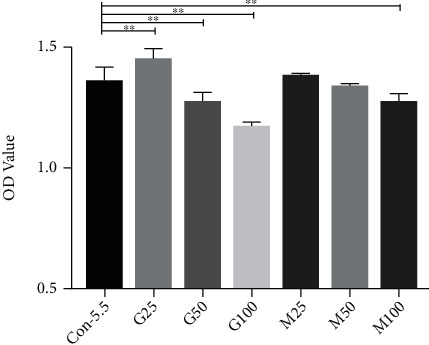
Cell proliferative ability of HRMECs using the CCK-8 assay. At 48 h after culturing, compared to the control group, increased cell proliferation at 48 was found in the G25 group, while the OD value was significantly lower in the G50 and G100 groups. No significant difference was observed between the hypertonic group and the control group. Con-5.5, G25-G100: concentration of glucose (5.5, 25, 50, and 100 mmol/l, respectively); M25-M100: concentration of M-mannitol (19.5, 44.5, and 94.5 mmol/l, respectively). CCK-8: Cell Counting Kit-8 assay. HRMEC: human retinal endothelial cell. OD value: optical density value. ^∗^*p* < 0.05; ^∗∗^*p* < 0.01.

We further investigate the CCK-8 assay and AI in the groups of 5.5 mM, 25 mM, 50 mM, and 100 mM to explore the extreme value of *in vitro* model of DR. We found that the proliferative capacity in the 50 mM is significantly lower compared to the 5.5 mM group (24 h: 0.91 ± 0.03 vs. 0.96 ± 0.45, *p* < 0.001; 48 h: 1.27 ± 0.05 vs. 1.36 ± 0.05, *p* < 0.001; and 72 h: 1.48 ± 0.06 vs. 1.58 ± 0.03, *p* = 0.001); furthermore, cell proliferation capacity was obviously inhibited in the 100 mM group (24 h: 0.87 ± 0.02 vs. 0.96 ± 0.45, *p* < 0.001; 48 h: 1.18 ± 0.02 vs. 1.36 ± 0.05, *p* < 0.001; and 72 h: 1.23 ± 0.01 vs. 1.58 ± 0.03, *p* < 0.001), which was not suitable for further study (Figure 1). In the TUNEL assay, the AI is significantly increased in the 25 mM glucose-treated group compared with the 5.5 mM group (19.89% ± 5.82% vs. 8.87% ± 3.09%, *p* = 0.024), and the increasing linear trend was found. In summary, the cell permeability, migration ability and AI of HRMEC showed an increasing trend, from 25 mM to 50 mM, while capacities were inhibited when glucose concentration increased further. 50 mM may be equal to the acute clinical value of diabetes and the extreme limit value in an *in vitro* study.

### 3.2. Cells Treated with 25 mM Have Higher Proliferative Capacity Compared to those Treated with 5.5 mM

The CCK-8 assay showed significant difference in OD values of each group after 24 h of culture (*F* = 25.567, *p* < 0.001). Compared to the control group, the OD in the G25 group was significantly higher (1.01 ± 0.05 vs. 0.96 ± 0.45 (control), *p* < 0.001), while it was significantly lower in the G50 and G100 groups (0.91 ± 0.03 vs. 0.96 ± 0.45, *p*_G50_ < 0.001; 0.87 ± 0.02 vs. 0.96 ± 0.45, *p*_G100_ < 0.001, respectively); in addition, no significant difference was observed between the hypertonic group and the control group.

Similar results were observed after 48 h of culturing (*F* = 79.037, *p* < 0.001). Compared to the control group, the OD in the G25 group was significantly higher (1.45 ± 0.04 vs. 1.36 ± 0.05, *p* < 0.001), while it was significantly lower in the G50 and G100 groups (1.27 ± 0.05 vs. 1.36 ± 0.05, *p* < 0.001; 1.18 ± 0.02 vs. 1.36 ± 0.05, *p* < 0.001, respectively). In addition, the M100 group was lower than the control group, and the difference was statistically significant (1.18 ± 0.02 vs. 1.36 ± 0.05, *p* < 0.001, respectively) (Figure 1).

After 72 h culturing, the overall difference of OD value between the groups was statistically significant (*F* = 61.144, *p* < 0.001). Compared to the control group, the OD in the G25 group was significantly higher (1.65 ± 0.12 vs. 1.58 ± 0.03, *p* = 0.006), while it was significantly lower in the G50 and G100 groups (1.48 ± 0.06 vs. 1.58 ± 0.03, *p* < 0.001; 1.23 ± 0.01 vs. 1.58 ± 0.03, *p* < 0.001, respectively). Compared with the control group, the M50 and M100 groups in the hypertonic group were significantly reduced (1.41 ± 0.02 vs. 1.58 ± 0.03, *p* < 0.001; 1.39 ± 0.07 vs. 1.58 ± 0.03, *p* < 0.001, respectively) (Figure 1). The results indicated that the better time point for studying HRMECs under higher glucose is at 48 h after culturing.

### 3.3. HRMECs Treated with 25 mM or 50 mM Have Higher Migration Capacity Compared to Those Treated with 5.5 mM (Transwell)

**Figure 2:**
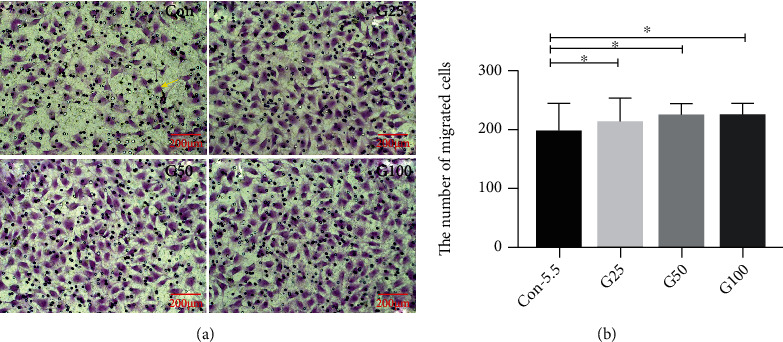
Cell migration ability detected by Transwell assay. (a) Representative images of migrated HRMECs treated with 5.5 mM, 25 mM, 50 mM, and 100 mM glucose, respectively, at 48 h. HRMEC cells were stained with 0.1% crystal violet (yellow, arrows). (b) The number of migrated HRMEC was significant higher in the 25 mM (238 ± 29 vs. 200 ± 42, *p* = 0.026), 50 mM (230 ± 16 vs. 200 ± 42, *p* = 0.021), and 100 mM (228 ± 18 vs. 200 ± 42, *p* = 0.031) groups in comparison with the 5.5 mM groups. Scale bar: 200 *μ*m (a). HRMEC: human retinal vascular endothelial cells; Con: control group (5.5 mmol/l); G25: 25 mmol/l; G50: 50 mmol/l; G100: 100 mmol/l. ^∗^*p* < 0.05.

Significant difference of the migration cells was found between the different glucose concentration groups at 48 h by transwell assay (*F* = 4.412, *p* = 0.008). The migration ability in HRMECs using transwell assay revealed that there was a significantly increased number of migration cells in the 25 mM (238 ± 29 vs. 200 ± 42, *p* = 0.026), 50 mM (230 ± 16 vs. 200 ± 42, *p* = 0.021), and 100 mM groups (228 ± 18 vs. 200 ± 42, *p* = 0.031) in comparison with the control 5.5 mM group at 48 h (Figure 2).

After 24, 48, and 72 h, the cell migration rate of the control group was 35.84% ± 8.19%, 49.00% ± 11.43%, and 59.31% ± 13.29%, respectively. Compared to the control group, at 24 h of culture, higher cell migration rate was observed in the G25 and G50 groups (45.79% ± 5.28% vs. 35.84% ± 8.19%, *p* < 0.001; 46.13 ± 5.53% vs. 35.84% ± 8.19%, *p* < 0.001), while it was lower in the G100 and M100 groups (25.54% ± 3.38% vs. 35.84% ± 8.19%, *p* < 0.001; 20.39% ± 7.62% vs. 35.84% ± 8.19%, *p* < 0.001). A similar effect was observed at 48 and 72 h post culture (all *p* < 0.05).

### 3.4. Apoptosis Indexes in Cells Treated with 25 mM or 50 mM Compared to Those Treated with 5.5 mM in HRMEC

PI/Hoechst staining and ImageJ were used to quantify the live and dead cells. We found that the apoptosis indexes of 5.5 mM groups, G25, G50, G100, M25, M50, and M100 groups were 7.71% ± 0.80%, 10.67% ± 2.06%, 11.06% ± 0.47%, 5.04% ± 0.40%, 8.07% ± 0.60%, 8.93% ± 0.56%, and 5.98% ± 0.33%, respectively, and the overall difference between the two groups was statistically significant (*F* = 51.101, *p* < 0.001) after 24 h of cell culturing. The apoptosis indexes of the G25 and G50 groups were significantly higher compared to the control group (10.67% ± 2.06% vs. 7.71% ± 0.80%, *p* < 0.001; 11.06% ± 0.47% vs. 7.71% ± 0.80%, *p* < 0.001).

At 48 h, the cell apoptosis indexes of 5.5 mM groups, G25, G50, G100, M25, M50, and M100 groups were 11.16% ± 0.84%, 20.84% ± 0.44%, 18.50% ± 0.88%, 11.19% ± 0.60%, 11.87% ± 0.40%, 15.55% ± 0.81%, and 14.15% ± 0.43%, respectively. The overall difference between the groups was statistically significant (*F* = 297.784, *p* < 0.001). The apoptosis indexes of G25, G50, and M50 groups were significantly higher compared to the control group, and the difference was statistically significant (20.84% ± 0.44% vs. 11.16% ± 0.84%, *p* < 0.001; 18.50% ± 0.88% vs. 11.16% ± 0.84%, *p* < 0.001; and 15.55% ± 0.81% vs. 11.16% ± 0.84%, *p* < 0.001).

At 72 h, the AI of 5.5 mM, G25, G50, G100, M25, M50, and M100 groups was 16.06% ± 0.42%, 27.02% ± 0.96, 25.56% ± 0.96%, 20.15% ± 0.64%, 17.97% ± 1.17%, 20.86% ± 0.68%, and 21.72% ± 0.81%, respectively. The overall difference between the groups was statistically significant (*F* = 194.267, *p* < 0.001). The apoptosis indexes of the G25, G50, M25, M50, and M100 groups were significantly higher than that in the control group, and the difference was statistically significant (*p* < 0.001).

**Figure 3:**
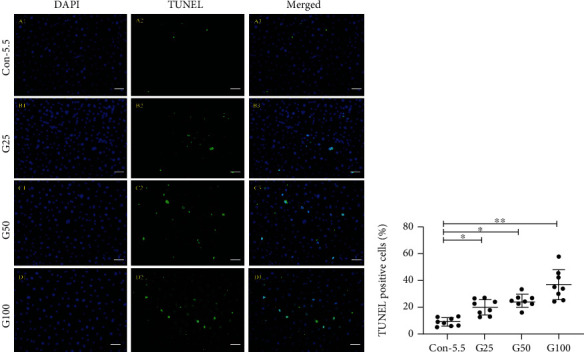
Representative fluorescence images and quantification of TUNEL-positive HRMECs. TUNEL staining (green, A2-D2), DAPI staining (blue, A1-D1), and merged image of HRMECs with different treatments. Merged images are presented as A3-D3. Con-5.5, G25-G100: concentration of glucose (5.5, 25, 50, and 100 mmol/l, respectively). HRMECs: human retinal microvascular endothelial cells. Scale bar: 200 *μ*m. ^∗^*p* < 0.05; ^∗∗^*p* < 0.01.

The TUNEL analysis further confirmed the PI/Hoechst result. The AI of the HRMECs in the 25 mM, 50 mM, and 100 mM is significantly higher than that in the control group (*p*_25mM_ = 0.024, *p*_50mM_ = 0.001, and *p*_100mM_ < 0.001) at 48 h (Figure 3).

### 3.5. HRMEC Treated with Hyperglycemia Groups Poses Higher Permeability Compared to Those Treated with 5.5 mM

After 24 h of cell culturing, the cell permeability of 5.5 mM, G25, G50, G100, M25, M50, and M100 groups was 8.12% ± 0.56%, 8.42% ± 0.23%, 8.82% ± 0.26%, 8.83% ± 0.25%, 8.39% ± 0.24%, 8.47% ± 0.25%, and 8.52% ± 0.27%, respectively. Cell permeability slightly increased with the increase of glucose concentration. In addition, no significant effect on cell permeability was observed in the mannitol-control groups.

### 3.6. The Angiogenic Ability of HRMEC Is Higher in the 25 mM and 50 mM Groups Compared to the 5.5 mM Group

**Figure 4:**
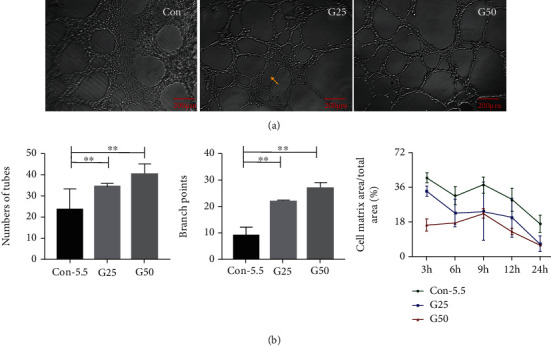
A Matrigel-based tube formation assay to assess the vasogenic activity of HRMEC. G25/G50 showed a stimulatory effect on angiogenic tube formation in the Matrigel assay. (a) HREMCs were cultured on Matrigel, and the cumulative numbers of circle-like structures (yellow arrow) and branch points were measured after 9 h. (b) The vasculogenic capacity was quantified in cells treated with different groups. Con-5.5, G25, and G50: concentration of glucose (5.5, 25, and 50 mmol/l, respectively). Tube formation assay was examined under an inverted fluorescence microscope with a ×10 objective. HRMEC: human retinal vascular endothelial cells. Scale bar: 200 *μ*m (a). ^∗^*p* < 0.05; ^∗∗^*p* < 0.01.

Angiogenesis was evaluated by a Matrigel-based tube formation assay and protein expression analysis of VEGFA in different groups. The number of tubes at 9 hours increased with the concentration of glucose (5.5 mM: 23.5 ± 9.87, 25 mM: 34.33 ± 1.51; and 50 mM: 40 ± 5.02), respectively (*F* = 10.124, *p* = 0.002), showing a statistical significance among the groups. The number of branch points was also significantly increased in the higher glucose groups (5.5 mM: 9 ± 3.16, 25 mM: 21.67 ± 0.52, and 50 mM: 26.67 ± 1.97, respectively, *F* = 105.613, *p* < 0.001). The area of the extracellular (tube metrics area) in the G25 and G50 groups were significantly less than that in the 5.5 mM group (*p*_G25_ = 0.013, *p*_G50_ = 0.024) (Figure 4).

ELISA was used to detect VEGFA secretion from HRMECs into the medium. The results showed that the amount of VEGFA secreted increased with increasing glucose concentrations (*F* = 9.000, *p* = 0.016).

### 3.7. The Biological Behavior of RGCs under Hyperglycemia Condition

**Figure 5:**
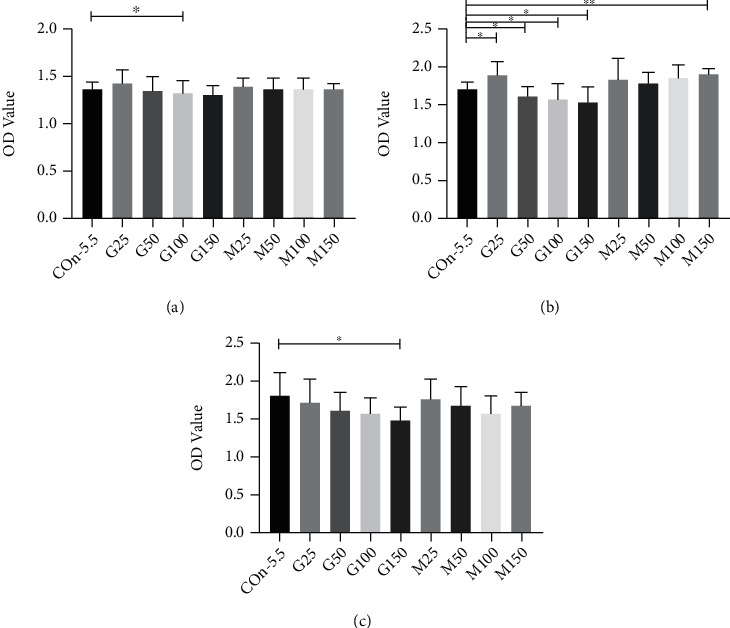
Cell viability of RCGs by the CCK-8 assay. Compared to the control group, the OD value was significantly lower in the G50, G100, and G150 at 48 h. (a–c) Cell proliferation ability in RGCs treated with different groups at 24, 48, and 72 h. Con-5.5, G25-G150: concentration of glucose (5.5, 25, 50, 100, and 150 mmol/l, respectively); M25-M150: concentration of M-mannitol (19.5, 44.5, 94.5, and 133.5 mmol/l, respectively). ^∗^*p* < 0.05; ^∗∗^*p* < 0.01. RGCs: retinal ganglion cells CCK-8: Cell Counting Kit-8 assay OD value: optical density value.

The cell variability of RGCs was detected by the CCK-8 assay. The OD value between the 5.5 mM, G25, G50, G100, G150, M25, M50, M100, and M150 groups was statistically significant (*F* = 8.041, *p* < 0.001) at 48 h. The OD value was significantly higher in the 25 mM (1.871 ± 0.218 vs. 1.718 ± 0.100, *p* = 0.023), while it was lower in the 50 mM (1.607 ± 0.128 vs. 1.718 ± 0.100, *p* = 0.013), 100 mM (1.562 ± 0.215 vs. 1.718 ± 0.100, *p* = 0.019), and 150 mM (1.535 ± 0.185 vs. 1.718 ± 0.100, *p* = 0.002) in comparison with the control 5.5 mM at 48 h. Compared with the control group, the M25, M50, and M100 groups were not significantly reduced (*p*_M25_ = 0.240, *p*_M50_ = 0.416, and *p*_M100_ = 0.163) at 48 h. The same declined trend of OD value was also found at 24 h and 72 h (Figure 5).

RGCs are the earliest affected retinal neurons in the retina. The long axons of RGCs play an important role in transmitting visual information along with photoreceptors, horizontal cells, amacrine cells, and bipolar cells, from the retina to the brain [20, 21]. To further evaluate the effects of hyperglycemia, the axons, which are the sole retinal neuron projections, connect the RGC cell body and brain. It is defined as the longest neurite extending from the RGC cell body [22]. RGCs were labeled by neurons and RGC-specific markers TUJ1 and BRN3A, respectively, followed by the ImageJ quantitative analysis. The proportion of RGCs with neurite extensions in the 5.5 mM, G25, G50, G100, G150, M25, M50, M100, and M150 groups was statistically significant (*F* = 49.655, *p* < 0.001) at 48 h. Immunofluorescence staining revealed that the proportion of RGCs with neurite extensions was significantly lower in the 25 mM (33 ± 13 vs. 49 ± 5, *p* =0.007), 50 mM (21 ± 7 vs. 49 ± 5, *p* < 0.001), 100 mM (20 ± 7 vs. 49 ± 5, *p* < 0.001), and 150 mM (17 ± 6 vs. 49 ± 5, *p* < 0.001) groups in comparison with that in the control group (5.5 mM) at 48 h. Compared with the control group, the M25, M50, M100, and M150 groups in the hypertonic group were not significantly different (*p*_M25_ = 0.691, *p*_M50_ = 0.297, *p*_M100_ = 0.716, and *p*_M150_ = 0.356) (Table 1). Apoptosis in RGCs was detected and quantified by the PI/Hoechst fluorescent staining and TUNEL assay, followed by the ImageJ analysis. AI was found statistical significance in the different glucose concentration groups at 24 h, 48 h, and 72 h detected by the PI/Hoechst staining (*F*_24h_ = 27.648, *p* = 0.001; *F*_48h_ = 11.863, *p* < 0.001; and *F*_72h_ = 9.880, *p* < 0.001). Compared to the control group, the AI of the G50, G100, and G150 groups was significantly higher after 24 h of cell culturing (*p*_G50_ = 0.015, *p*_G100_ = 0.003, and *p*_G150_ = 0.001). AI in the G25, G100, and G150 groups was significantly higher than that in the control group at 48 h (*p*_G25_ = 0.006, *p*_G100_ = 0.006, and *p*_G150_ = 0.002). The AI of the G50, G100, and G150 groups was significantly higher than that of the control group at 72 h (*p*_G50_ = 0.003, *p*_G100_ = 0.016, and *p*_G150_ = 0.001). In addition, the AI of hypertonic groups was not significantly higher than that in the 5.5 mM group at 24 h, 48 h, and 72 h (all *p* > 0.05) (Table 2).

TUNEL assay further confirmed the above results. The AI of the RGCs in the 50 mM, 100 mM, and 150 mM is significantly higher than that in the control group (*p*_50mM_ < 0.001, *p*_100mM_ < 0.001, and *p*_150mM_ < 0.001) at 48 h (Table 3).

## 4. Discussion

In this study, we have optimized glucose concentrations to model diabetic retinal endothelial (25-50 mM) or neuronal (50-150 mM) dysfunction *in vitro*, which have a wide range of downstream applications.

*In vitro* studies have been colloquially named “test-tube” experiments, providing a convenient, time- or cost-consuming, specific, and more detailed analysis which cannot be done within the whole organism. The advantages of *in vitro* study are simplicity [22–24], species specificity [25], convenience and automation [26]. *In vitro* studies are essential to explore the pathophysiology of retinal damage by hyperglycemia [27, 28]. Immortalized cell lines and primary isolated retinal endothelial and ganglion cells are currently the most widely used *in vitro* models for exploring the pathogenesis of DR, a neuronal vascular disorder [8].

Currently, the commercially available *in vitro* model of DR in different cell types is 5.5 mM (as the normal control) and 25 mM (as the higher glucose) for HRMEC and 35 mM for RGCs, respectively [29–31]. However, there is no evidence to prove that 25 mM or 30 mM-35 mM glucose *in vitro* model is simulation to the physiological conditions (hyperglycemia) of the human body, and 25 mM should not be considered the upper limit of *in vitro* research. In this study, we found that concentrations of 25 mM to 50 mM glucose could induce the proliferation of HRMEC, promote cell migration capacity, and improve angiogenic ability (tube formation), promoting cell apoptosis (from 25 mM to 100 mM). All of the above results indicate that 25 mM is a cutoff value, and 25-50 mM is the proper concentration to stimulate diabetic microvasculopathy. This concentration range also can be used to study retinal neovascularization in proliferative retinopathy. Furthermore, under the hyperglycemia pathological condition, the AI of HRMEC was significantly increased, indicating that a high concentration of glucose may increase the oxidative stress within the cell, activating the signal transduction pathway, promoting cell apoptosis. Leal et al. [32] found that the number of apoptotic cells significantly increased after HRMEC was treated with 30 mM glucose, consistent with our result.

On the other hand, the concentration of 50 mM to 150 mM glucose exhibits inhibition on the cell variability of RGCs at 24 h, 48 h, and 72 h. Immunofluorescence staining revealed that the proportion of RGCs with neurite extensions was significantly lower in the 50 mM, 100 mM, and 150 mM groups than that in the control group (5.5 mM) at 48 h. The TUNEL assay also found that AI is significantly higher in the 50 mM, 100 mM, and 150 mM groups, indicating 50 mM may be taken as the cutoff value for *in vitro* study of neuronal degeneration under hyperglycemia, and 50 mM-100 mM may be the proper concentration to stimulate diabetic microvasculopathy.

In the present study, we found that glucose concentration was positively correlated with the cell permeability of HRMEC. This may due to the decreased expression levels of cell tight junction proteins [33]. Extremely high glucose concentrations (100 mM) can significantly inhibit cell proliferation. However, we cannot ignore the effect of osmotic pressure on cell biological behavior at this concentration. These results suggest that the extremely high glucose concentrations may not be suitable for *in vitro* testing. By contrast, considering the behavior of the HRMEC under different glucose concentrations, 25 mM-50 mM may be of the appropriate range and can be further applied in further *in vitro* study such as drug interventions for DR.

HRMEC has an important role in the maintenance of the normal function of RBB in human. Its pathological changes are the basis of diabetic microvasculopathy [34]. Our study investigated the gradient of glucose concentration and osmotic pressure on the biological behavior of HRMEC *in vitro*. The results have provided new insight into this area.

Previous studies have found a significantly lower cell proliferation rate in cells treated with a glucose concentration of 25 mM compared to cells treated with 5.5 mM [32, 35]. However, Sun et al. found that 25 mM glucose concentration promoted cell proliferation, migration, and apoptosis, consistent with our findings [36]. In our study, we have shown that increased osmotic pressure does not affect the biological behavior of HRMEC, which was consistent with previous studies that osmotic pressure did not affect cell viability at all, but some studies concluded that osmotic pressure of 25 mM had a negligible effect on the biological behavior of HRMEC [37–39].

The best concentration of D-glucose to use in human cell cultures to mimic the diabetic condition is yet to be set up. Furthermore, the concentration of D-glucose concentration will vary accordingly based on different cell lines. In the current study, we investigated for the first time the effects of the concentration gradient of glucose and osmotic pressure on the biological behavior of HRMEC, providing the rational concentration of D-glucose to mimic the DR condition. We found that 25 mM (the cutoff value) to 50 mM (the extreme value) is a suitable high glucose concentration.

To further analyze the effects and mechanisms of high sugar on HREMC biological behavior, we carried out a tube formation assay, a fast, quantifiable method to detect the angiogenic activity of HRMEC [40]. The advantages of this assay are that it is relatively easy to set up, requires a short culture period, is quantifiable, and is amenable to high-throughput analysis. HRMECs treated with 25 mM or 50 mM glucose have a larger angiogenic capacity than those treated with 5.5 mM glucose. This result suggested a potential biological basis of abnormal neovascularization in DR patients. We also found that the quantification results of the tube formation assay are concomitant with the elevated protein expression of VEGFA. This finding suggested that VEGFA may be the key regulator for the high glucose-induced angiogenesis process and established the rationality of 25 mM-50 mM glucose concentration for the *in vitro* hyperglycemia model.

In this study, we found that the cell variability of RGCs is significantly lower in the 50 mM to 150 mM and the linear declined trend was evidence from 5.5 mM to 150 mM, which is consistent with previous studies [41]. RGCs were further labeled with neurons and RGC-specific markers TUJ1 and BRN3A, respectively, followed by the ImageJ quantitative analysis [42]. Immunofluorescence staining revealed that the proportion of RGCs with neurite extensions was significantly lower in the 50 mM (*p* < 0.05), 100 mM (*p* < 0.05), and 150 mM (*p* < 0.05) groups in comparison with that in the control group (5.5 mM) at 48 h. the AI is significantly higher in the 50 mM (*p* < 0.001), 100 mM (*p* < 0.001), and 150 mM (*p* < 0.001) groups compared to the control 5.5 mM at 48 h. The results indicate that 50 mM-100 mM may be used as the hyperglycemia *in vitro* model, and 50 mM and 150 mM may be taken as the cutoff and extreme value.

In this study, we set the hyperosmotic control group by artificially changing the osmotic pressure in the cell culture medium: Mannitol was added to the 5.5 mM group to adjust the osmotic pressure to correspond with the same osmotic pressure of G25, G50, G100, and G150, thus forming M25, M50, M100, and M150 (mannitol concentrations 19.5, 44.5, 94.5, and 133.5 mmol/l, respectively). The osmosis pressure of the control group (Con-5.5) was about 320 mOsm. According to the permeable pressure calculation formula, it can be obtained that the remaining groups of osmosis pressure were approximately 340 mOsm (G25/M25), 365 mOsm (G50/M50), 415 mOsm (G100/M100), and 465 mOsm (G150/M150). In our study, we have shown that increased osmotic pressure does not affect the biological behavior of RGCs from 25 mM to 50 mM.

Our previous studies have shown that neurodegeneration is an earlier pathological change in the retina than microvasculopathy. It is interesting to study further the interactions between retinal endothelium cells and neurons. This study provides a basis for the next step in exploring how gene transfection can rescue vascular disease and early neuropathy. As the Müller glia are also involved in DR and are a component in the neuronal vascular unit, further studies warrant setting up more complicated *in vitro* models to stimulate the retinal neural vascular unit.

We used the healthy HRMECs and RGCs for establishing the *in vitro* model of DR. As the endothelial and neuronal cells in advanced DR stages may react differently to the 25-50 mM or 50-100 mM of glucose, respectively, this *in vitro* model may more applicable to stimulate the early stage of DR or even the preclinical stage of DR. This *in vitro* model also represents a valid tool useful for pathological investigation, drug screenings, and gene therapy of DR.

US-FDA approved Voretigene neparvovec-rzyl (Luxturna) gene therapy in 2017 to rescue the apoptotic photoreceptors in Leber's congenital amaurosis [43, 44]. This led to the bright prospects of gene therapy on multigene eye diseases, including DR. Currently, 12 serotypes of adeno-associated virus have been applied as gene vectors. It is necessary to test the cell-specific serotype *in vitro*, especially in pathological conditions (high glucose) first [45]. Therefore, the establishment of proper hyperglycemia *in vitro* model is essential for further study of DR.

Due to the complexity of the internal environment and the diversity of the microenvironment, the application of animal models for investigating diabetic retinopathy faces great limitations and challenges. Therefore, the *in vitro* study could provide insight for studying the pathogenesis, progression, and even drug intervention of retinal diseases.

In conclusion, glucose of 25 mM to 50 mM is the appropriate range and 100 mM extreme value of hyperglycemia for HRMEC *in vitro*; 50 mM and 150 mM are the proper range for RGCs. The impact of osmotic in higher glucose can be omitted. High glucose-induced VEGF is the key pathogenic factor of angiogenesis in endothelial cells.

## Figures and Tables

**Table 1:** Neurite extensions in RGCs detected by immunofluorescence staining at 48 h.

Groups	The proportion of RGCs with neurite extensions (%)	*p* value
Con	49 ± 5	—
*G25*	33 ± 13	*0.007^a^* ^∗^
*G50*	21 ± 7	*<0.001^a^* ^∗^
*G100*	20 ± 7	*<0.001^a^* ^∗^
*G150*	17 ± 6	*<0.001^a^* ^∗^
M25	51 (42-55)	0.691^b^
M50	43 ± 15	0.297^b^
M100	50 ± 13	0.716^a^
M150	46 ± 9	0.356^a^

^∗^Statistically significant: ^∗^*p* < 0.05. According to the type of data and the data distribution, ^a^independent-sample *t*-test and the ^b^Mann-Whitney *U* test were applied. Con-5.5, G25-G150: concentration of glucose (5.5, 25, 50, 100, and 150 mmol/l, respectively); M25-M150: concentration of M-mannitol (19.5, 44.5, 94.5, and 133.5 mmol/l, respectively). RGCs: retinal ganglion cells.

**Table 2:** Apoptotic index in RGCs at 24 h, 48 h, and 72 h.

Groups	24 h	*p* value	48 h	*p* value	72 h	*p* value
	Apoptotic index		Apoptotic index		Apoptotic index	
Con	0.042 (0.028-0.047)	—	0.013 ± 0.007		0.091 ± 0.031	—
G25	0.069 (0.031-0.112)	0.171^b^	0.024 ± 0.007	0.006^a^	0.132 ± 0.083	0.182^a^
*G50*	0.108 (0.071-0.144)	0.015^b^	0.018 ± 0.009	0.284^a^	0.225 ± 0.095	*0.003^a^* ^∗^
*G100*	0.120 (0.061-0.256)	0.003^b^	0.025 ± 0.008	0.006^a^	0.181 ± 0.088	*0.016^a^* ^∗^
*G150*	0.148 (0.079-0.184)	0.001^b^	0.055 ± 0.027	0.002^a^	0.291 ± 0.124	*0.001^a^* ^∗^
M25	0.049 (0.025-0.082)	0.691^b^	0.016 ± 0.003	0.329^a^	0.089 ± 0.024	0.893^a^
M50	0.036 (0.024-0.059)	0.691^b^	0.017 ± 0.004	0.221^a^	0.089 ± 0.030	0.856^a^
M100	0.050 (0.030-0.065)	0.354^b^	0.019 ± 0.004	0.054^a^	0.097 ± 0.040	0.733^a^
M150	0.056 (0.030-0.082)	0.270^b^	0.016 ± 0.008	0.455^a^	0.093 ± 0.031	0.918^a^

^∗^Statistically significant: ^∗^*p* <0.05. According to the type of data and the data distribution, ^a^independent-sample *t*-test and the ^b^Mann-Whitney *U* test were applied. Con-5.5, G25-G150: concentration of glucose (5.5, 25, 50, 100, and 150 mmol/l, respectively); M25-M150: concentration of M-mannitol (19.5, 44.5, 94.5, and 133.5 mmol/l, respectively).

**Table 3:** TUNEL-positive cells in RGCs using TUNEL staining at 48 h.

Groups	TUNEL-positive cells (%)	*p* value
Con	14.97 ± 7.48	—
G25	27.31 ± 2.41	0.364
*G50*	44.88 ± 7.31	*<0.001* ^∗^
*G100*	52.20 ± 2.86	*<0.001* ^∗^
*G150*	84.37 ± 2.43	*0.001* ^∗^

^∗^ *p* value: the difference of the high-glucose groups (25 mM, 50 mM, 100 mM, and 150 mM) and the control group (5.5 mM) was analyzed with one-way analysis of variance.

## Data Availability

All data generated or analyzed during this study are included in this published article.

## Abbreviations

AI: Apoptosis index
BRB: Blood retinal barrier
BSA: Bovine serum albumin
CCK-8: Cell Counting Kit-8
DMEM: Dulbecco's modified Eagle's medium
DR: Diabetic retinopathy
ELISA: Enzyme-linked immunosorbent assay
FITC: Fluorescein Isothiocyanate
FBS: Fetal bovine serum
HBSS: Hank's balanced salt solution
HRMEC: Human retinal microvascular endothelial cell
OD: Optical density
PBS: Permeabilization solution
PI: Propidium iodide
RMEC: Retinal microvascular endothelial cell
RGCs: Retinal ganglion cells
TUJ1: Antineuronal class III *β*-tubulin
VEGFA: Vascular endothelial growth factor-A.

## Supplementary Materials

Supplementary Materials Supplemental Figure 1: cell migration ability detected by scratch assay. Compared to the control group, at 24 h of culture, higher cell migration rate was observed in the G25 and G50 groups, while lower in the G100 and M100 groups. Similar effect was observed at 48 (*p* < 0.05) and 72 h (*p* < 0.01). (A1-A7) The images of the scratches at 0 h, 24 h (A8-A14), 48 h (A15-A21), and 72 h (A22-A28). (B1-B3) Cell migration rate in cells treated with different groups at 24, 48, and 72 h. Con-5.5, G25-G100: concentration of glucose (5.5, 25, 50, and 100 mmol/l, respectively); M25-M100: concentration of M-mannitol (19.5, 44.5, and 94.5 mmol/l, respectively). Scratch assay was examined under an inverted fluorescence microscope with a ×4 objective. Scale bar: 200 *μ*m (A). ^∗^*p* < 0.05; ^∗∗^*p* < 0.01. Supplemental Figure 2: representative composite images showing morphological changes of selected HREMCs detected with dual staining of Hoechst33342/PI. Green arrows: (1) viable cells with normal nuclei and (2) cells with apoptotic nuclei which was labeled by Hoechst33342. White arrows: (1) dead cells with normal nuclei and (2) dead cells with apoptotic nuclei which was labeled by PI. Yellow arrows: dead cells with apoptotic nuclei which were labeled by PI and Hoechst33342. Con-5.5, G25-G100: concentration of glucose (5.5, 25, 50, and 100 mmol/l, respectively); M25-M100: concentration of M-mannitol (19.5, 44.5, and 94.5 mmol/l, respectively). Cells were imaged by fluorescence microscope (magnification ×100). AI: apoptosis index: the number of PI-positive cells/the number of Hoechst-stained cells. HRMEC: human retinal vascular endothelial cells. PI: propidium iodide. Scale bar: 200 *μ*m (A). ^∗^*p* < 0.05; ^∗∗^*p* < 0.01. Supplemental Figure 3: HRMEC permeability evaluated by the FITC-labeled BSA method in different groups. After 24 h of cell culture, cell permeability slightly increased with the increase of glucose concentration. No significant effect on cell permeability was observed in the mannitol-control groups. Con-5.5, G25-G100: concentration of glucose (5.5, 25, 50, and 100 mmol/l, respectively); M25-M100: concentration of M-mannitol (19.5, 44.5, and 94.5 mmol/l, respectively). ^∗^*p* < 0.05; ^∗∗^*p* < 0.01. Supplemental Figure 4: the protein expression analysis of VEGFA in different groups using ELISA. Compared to the control group, increased VEGF expression at 24, 48, and 72 h was found in the G25 group. (A–C) VEGFA expression in HRMECs treated with different groups at 24, 48, and 72 h. Con-5.5, G25, and G50: concentration of glucose (5.5, 25, and 50 mmol/l, respectively). VEGFA: vascular endothelial growth factor-A. ELISA: enzyme-linked immunosorbent assay. ^∗^*p* < 0.05; ^∗∗^*p* < 0.01. Supplemental Figure 5: representative immunofluorescence images of RGCs in different groups at 48 h. RGCs were positively stained with TUJ1 (green, A1-A9, yellow arrows), DAPI (blue, B1-B9, white arrows), and Brn3a (red, C1-C9, green arrows) which are neuronal-specific, nucleus, and RGC-specific markers, respectively. Merged images were presented as D1-D9. Con-5.5, G25-G150: concentration of glucose (5.5, 25, 50, 100, and 150 mmol/l, respectively); M25-M150: concentration of M-mannitol (19.5, 44.5, 94.5, and 133.5 mmol/l, respectively). RGCs: retinal ganglion cells. Scale bar: 200 *μ*m (A). ^∗^*p* < 0.05; ^∗∗^*p* < 0.01. Supplemental Figure 6: apoptotic index of RGCs in different groups detected and quantified by the PI/Hoechst33342 fluorescence staining. RGCs were cultured and stained by the PI/Hoechst33342 fluorescence staining at 24 h (A1-A9), 48 h (A10-A18), and 72 h (A19-A27). (B1-B3) AI in cells treated with different groups at 24, 48, and 72 h. Quantification of apoptotic RGCs at 24 h (B1), 48 h (B2), and 72 h (B3) by the ImageJ analysis. Compared to the control group, the AI of the G50, G100, and G150 groups was significantly higher after 24 h of cell culturing (*p* < 0.05). At 48 h, the AI in the G50, G100, and G150 groups was significantly higher compared to that in the control group (*p* < 0.05). At 72 h, the AI of the G50, G100, and G150 groups was significantly higher than that of the control group (*p* < 0.05). Green arrow indicates (1) viable cells with normal nuclei and (2) cells with apoptotic nuclei which was labeled by Hoechst33342. Yellow arrow indicates (1) dead cells with normal nuclei and (2) dead cells with apoptotic nuclei which was labeled by PI. White arrow indicates dead cells with apoptotic nuclei which was labeled by PI and Hoechst33342. Con-5.5, G25-G150: concentration of glucose (5.5, 25, 50, 100, and 150 mmol/l, respectively); M25-M150: concentration of M-mannitol (19.5, 44.5, 94.5, and 133.5 mmol/l, respectively). RGCs: retinal ganglion cells, AI: apoptosis index: the number of PI-positive cells/the number of Hoechst-stained cells. Cells were imaged by fluorescence microscope (magnification ×100). Scale bar: 200 *μ*m (A). ^∗^*p* < 0.05; ^∗∗^*p* < 0.01. Supplemental Figure 7: representative immunofluorescence images and quantification of TUNEL-positive cells. TUNEL staining (green, A2-E2), DAPI staining (blue, A1-E1), and merged image of RGCs with different treatments. Merged images were presented as A3-E3. Con-5.5, G25-G150: concentration of glucose (5.5, 25, 50, 100, and 150 mmol/l, respectively). Scale bar: 200 *μ*m (A). ^∗^*p* < 0.05; ^∗∗^*p* < 0.01.
