# Supplementary material for: An In Vitro Model of Diabetic Retinal Vascular Endothelial Dysfunction and Neuroretinal Degeneration
Source: J Diabetes Res. 2021 Nov 10;2021:9765119. doi: 10.1155/2021/9765119 (PMC8598328; doi:10.1155/2021/9765119)
Supplement: Supplementary Materials — Supplemental Figure 1: cell migration ability detected by scratch assay. Compared to the control group, at 24 h of culture, higher cell migration rate was observed in the G25 and G50 groups, while lower in the G100 and M100 groups. Similar effect was observed at 48 (p < 0.05) and 72 h (p < 0.01). (A1-A7) The images of the scratches at 0 h, 24 h (A8-A14), 48 h (A15-A21), and 72 h (A22-A28). (B1-B3) Cell migration rate in cells treated with different groups at 24, 48, and 72 h. Con-5.5, G25-G100: concentration of glucose (5.5, 25, 50, and 100 mmol/l, respectively); M25-M100: concentration of M-mannitol (19.5, 44.5, and 94.5 mmol/l, respectively). Scratch assay was examined under an inverted fluorescence microscope with a ×4 objective. Scale bar: 200 μm (A). ∗p < 0.05; ∗∗p < 0.01. Supplemental Figure 2: representative composite images showing morphological changes of selected HREMCs detected with dual staining of Hoechst33342/PI. Green arrows: (1) viable cells with normal nuclei and (2) cells with apoptotic nuclei which was labeled by Hoechst33342. White arrows: (1) dead cells with normal nuclei and (2) dead cells with apoptotic nuclei which was labeled by PI. Yellow arrows: dead cells with apoptotic nuclei which were labeled by PI and Hoechst33342. Con-5.5, G25-G100: concentration of glucose (5.5, 25, 50, and 100 mmol/l, respectively); M25-M100: concentration of M-mannitol (19.5, 44.5, and 94.5 mmol/l, respectively). Cells were imaged by fluorescence microscope (magnification ×100). AI: apoptosis index: the number of PI-positive cells/the number of Hoechst-stained cells. HRMEC: human retinal vascular endothelial cells. PI: propidium iodide. Scale bar: 200 μm (A). ∗p < 0.05; ∗∗p < 0.01. Supplemental Figure 3: HRMEC permeability evaluated by the FITC-labeled BSA method in different groups. After 24 h of cell culture, cell permeability slightly increased with the increase of glucose concentration. No significant effect on cell permeability was observed in the mannitol-co [file 9765119.f1.pdf]

Supplemental Figures and Figure Legends

A

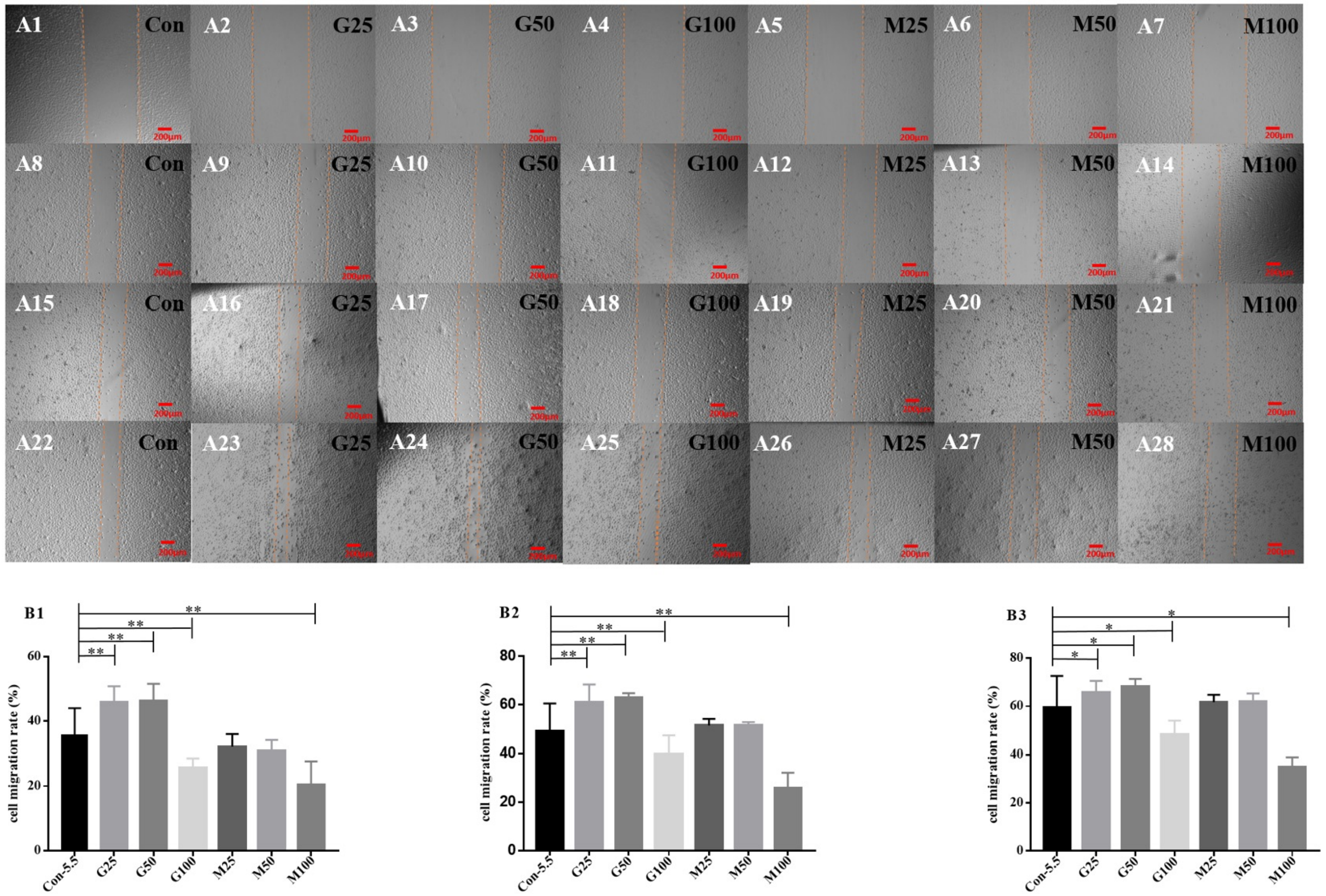

Supplemental Figure 1. Cell migration ability detected by scratch assay. Compared to the control group, at 24h of culture, higher cell migration rate was observed in the G25 and G50 groups, while lower in the G100 and M100 groups. Similar effect was observed at 48 ($P < 0.05$) and 72h ($P < 0.01$) A1-A7, the images of the scratches at 0h, 24h(A8-A14), 48h (A15-A21). and 72h (A22-A28). B1-B3, cell migration rate in cells treated with different groups at 24, 48 and 72h.Con-5.5, G25-G100: concentration of glucose (5.5,25,50 and 100mmol/l, respectively); M25-M100: concentration of M-mannitol (19.5, 44.5 and 94.5mmol/l, respectively). Scratch assay was examined under an inverted fluorescence microscope with a 4X objective. Scale bar: 200 μm(A). *$P < 0.05$; **$P < 0.01$

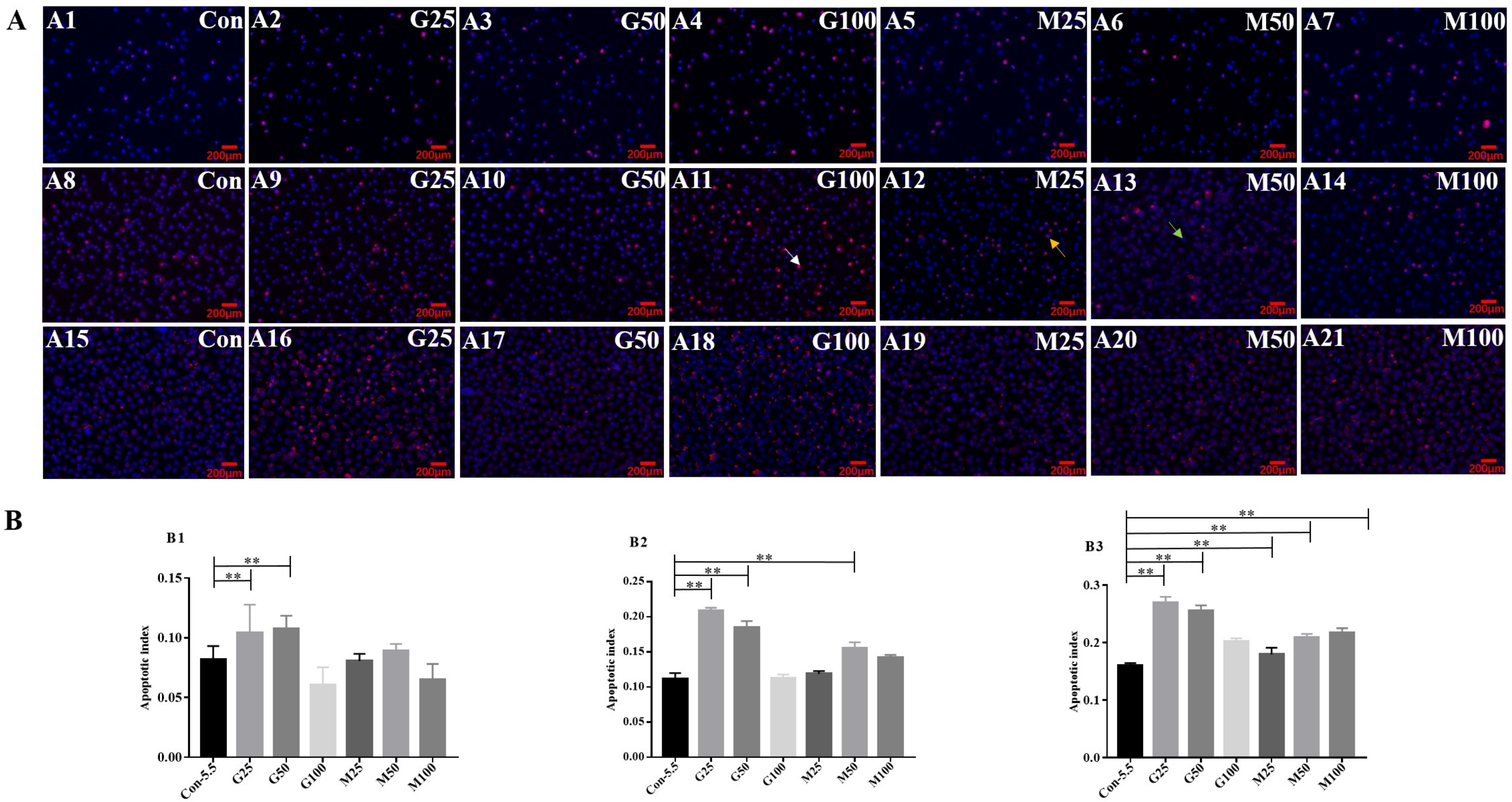

Supplemental Figure 2. Representative composite images showing morphological changes of selected HREMCs detected with dual staining of Hoechst33342 /PI. Green arrows (1) viable cells with normal nuclei (2) cells with apoptotic nuclei which was labeled by Hoechst33342; White arrows (1) dead cells with normal nuclei (2) dead cells with apoptotic nuclei which was labeled by PI; Yellow arrows: dead cells with apoptotic nuclei which were labeled by PI and Hoechst33342. Con-5.5, G25-G100: concentration of glucose (5.5, 25, 50 and 100mmol/l, respectively); M25-M100: concentration of M-mannitol (19.5, 44.5 and 94.5mmol/l, respectively). Cells were imaged by fluorescence microscope (magnification 100x). AI: Apoptosis index: the number of PI-positive cells/the number of Hoechst-stained cells. HRMEC: Human retinal vascular endothelial cells, PI: Propidium Iodide. Scale bar: 200 μm(A). *$P < 0.05$; **$P < 0.01$

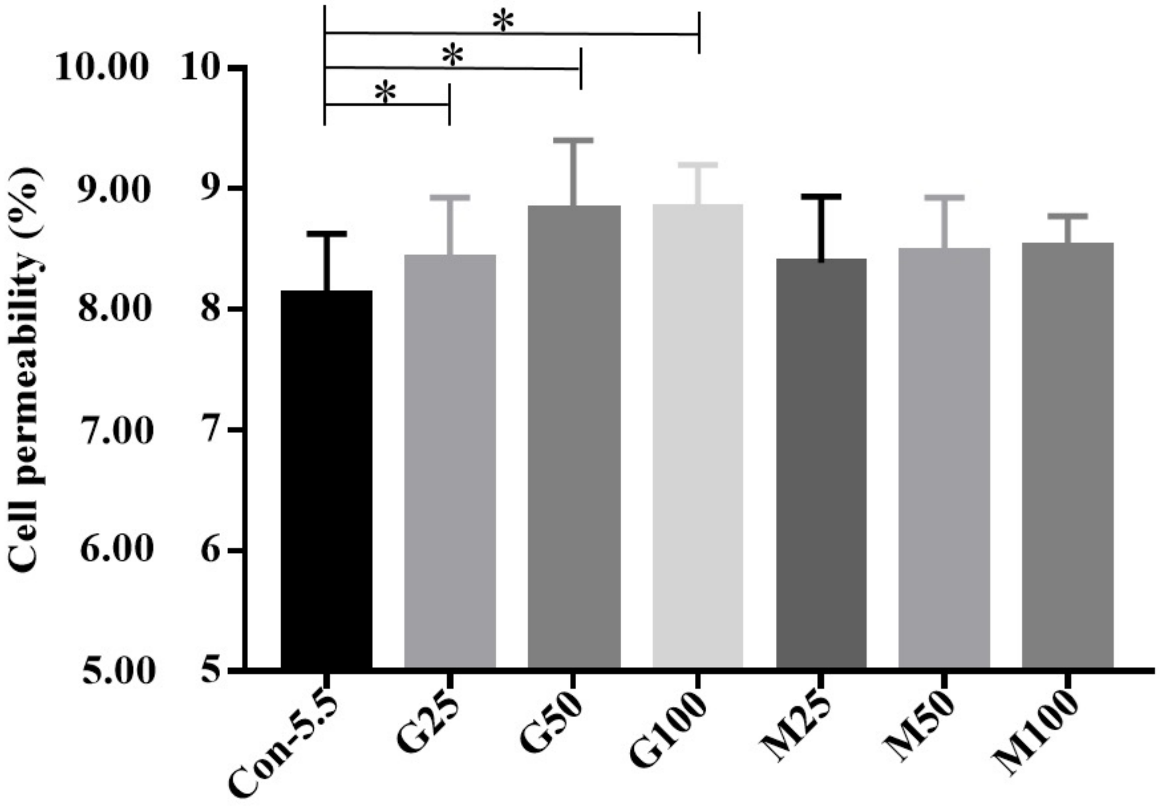

Supplemental Figure 3. HRMECs Permeable ability evaluated by FITC labeled BSA method in different groups. After 24 h of cell culture, cell permeability slightly increased with the increase of glucose concentration. No significant effect on cell permeability was observed in the mannitol-control groups. Con-5.5, G25-G100: concentration of glucose (5.5, 25, 50 and 100mmol/l, respectively); M25-M100: concentration of M-mannitol (19.5, 44.5 and 94.5mmol/l, respectively). **P* < 0.05; ***P* < 0.01

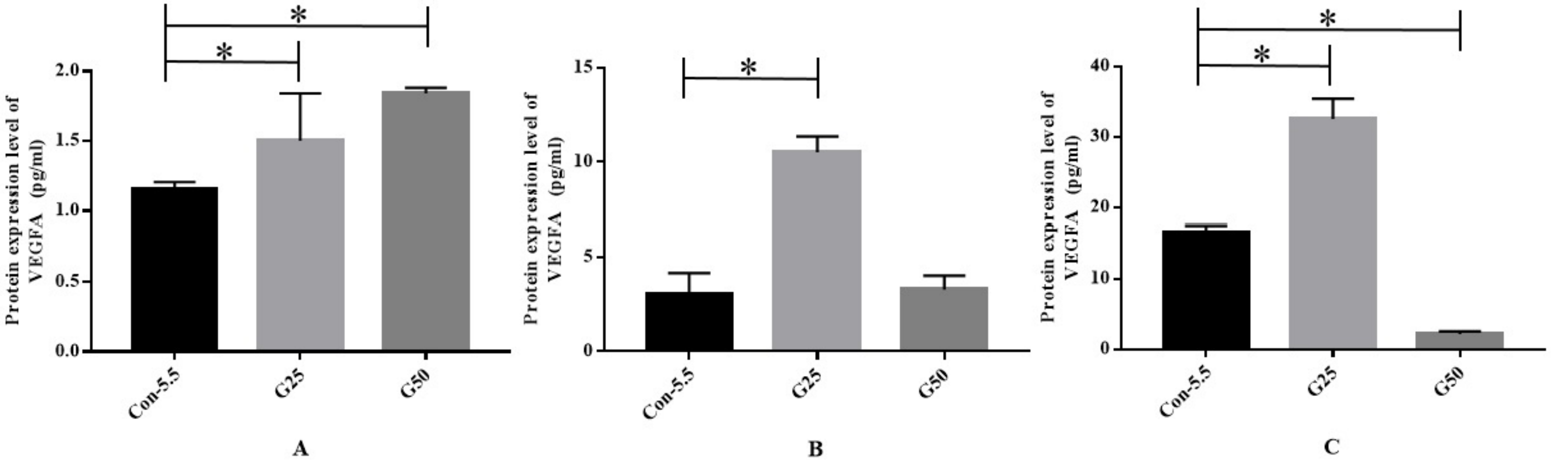

Supplemental Figure 4. The protein expression analysis of VEGFA in different groups using ELISA. Compared to the control group, increased VEGF expression at 24, 48 and 72 h was found in the G25 group. A, B, C, VEGFA expression in HRMECs treated with different groups at 24, 48 and 72h.Con-5.5, G25 and G50: concentration of glucose (5.5, 25 and 50 mmol/l, respectively). VEGFA: Vascular endothelial growth factor-A . ELISA: Enzyme-linked immunosorbent assay.**P* < 0.05; ***P* < 0.01.

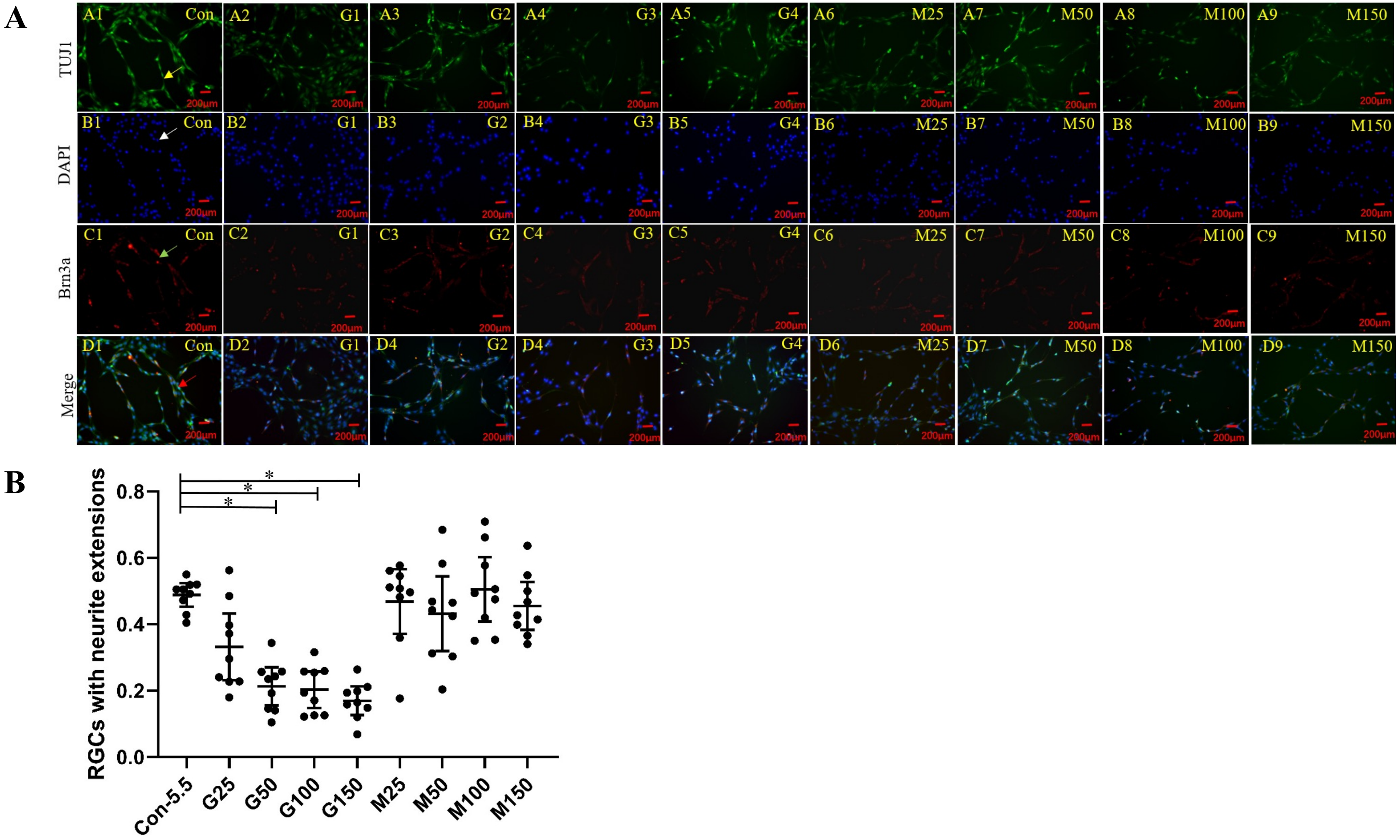

Supplemental Figure 5. Representative immunofluorescence images of RGCs in different groups at 48h. RGCs were positively stained with TUJ1(green, A1-A9, yellow arrows), DAPI (blue, B1-B9, white arrows) and Brn3a (red, C1-C9, green arrows) which are neuronal-specific, nucleus and RGC-specific markers, respectively. Merged images were presented as D1-D9. Con-5.5, G25-G150: concentration of glucose (5.5, 25, 50,100 and 150mmol/l, respectively); M25-M150: concentration of M-mannitol (19.5, 44.5,94.5 and 133.5mmol/l, respectively). RGCs: retinal ganglion cells. Scale bar: 200 μm (A). $*P < 0.05$; $**P < 0.01$

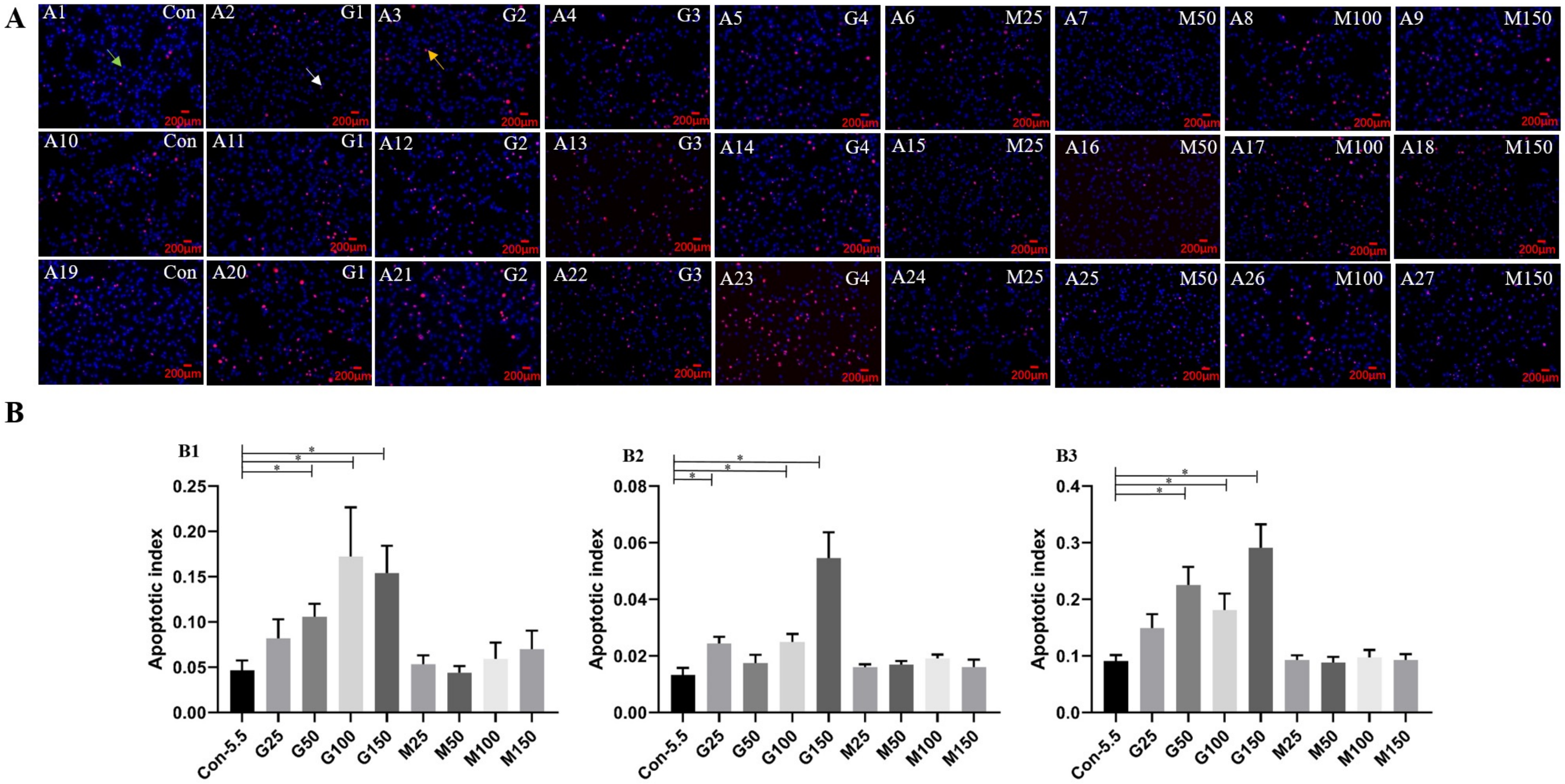

Supplemental Figure 6. Apoptotic index of RGCs in different groups detected and quantified by PI/Hoechst33342 fluorescence staining. RGCs were cultured and stained by PI/Hoechst33342 fluorescence staining at 24h (A1-A9), 48h(A10-A18) and 72h (A19-A27). B1-B3, AI in cells treated with different groups at 24,48 and 72h. Quantification of apoptotic RGCs at 24h (B1), 48h (B2) and 72h (B3) by image J analysis. Compared to the control group, the AI of the G50, G100 and G150 groups were significantly higher after 24h of cell culturing ($P<0.05$). At 48 h, AI in the G50, G100 and G150 groups were significantly higher compared to the control group ($P < 0.05$). At 72 h, the AI of the G50, G100 and G150 groups were significantly higher than control group ($P < 0.05$). Green arrow indicates (1) viable cells with normal nuclei(2) cells with apoptotic nuclei which was labeled by Hoechst33342; Yellow arrow indicates (1) dead cells with normal nuclei (2) dead cells with apoptotic nuclei which was labeled by PI; White arrow indicates: dead cells with apoptotic nuclei which was labeled by PI and Hoechst33342.Con-5.5, G25-G150:concentration of glucose (5.5, 25, 50,100 and 150mmol/l, respectively); M25-M150:concentration of M-mannitol (19.5, 44.5,94.5 and 133.5mmol/l, respectively). RGCs: retinal ganglion cells, AI: Apoptosis index: the number of PI-positive cells/the number of Hoechst-stained cells. PI: Propidium Iodide. Cells were imaged by fluorescence microscope (magnification 100x). Scale bar: 200 μm (A). $*P < 0.05$; $**P < 0.01$

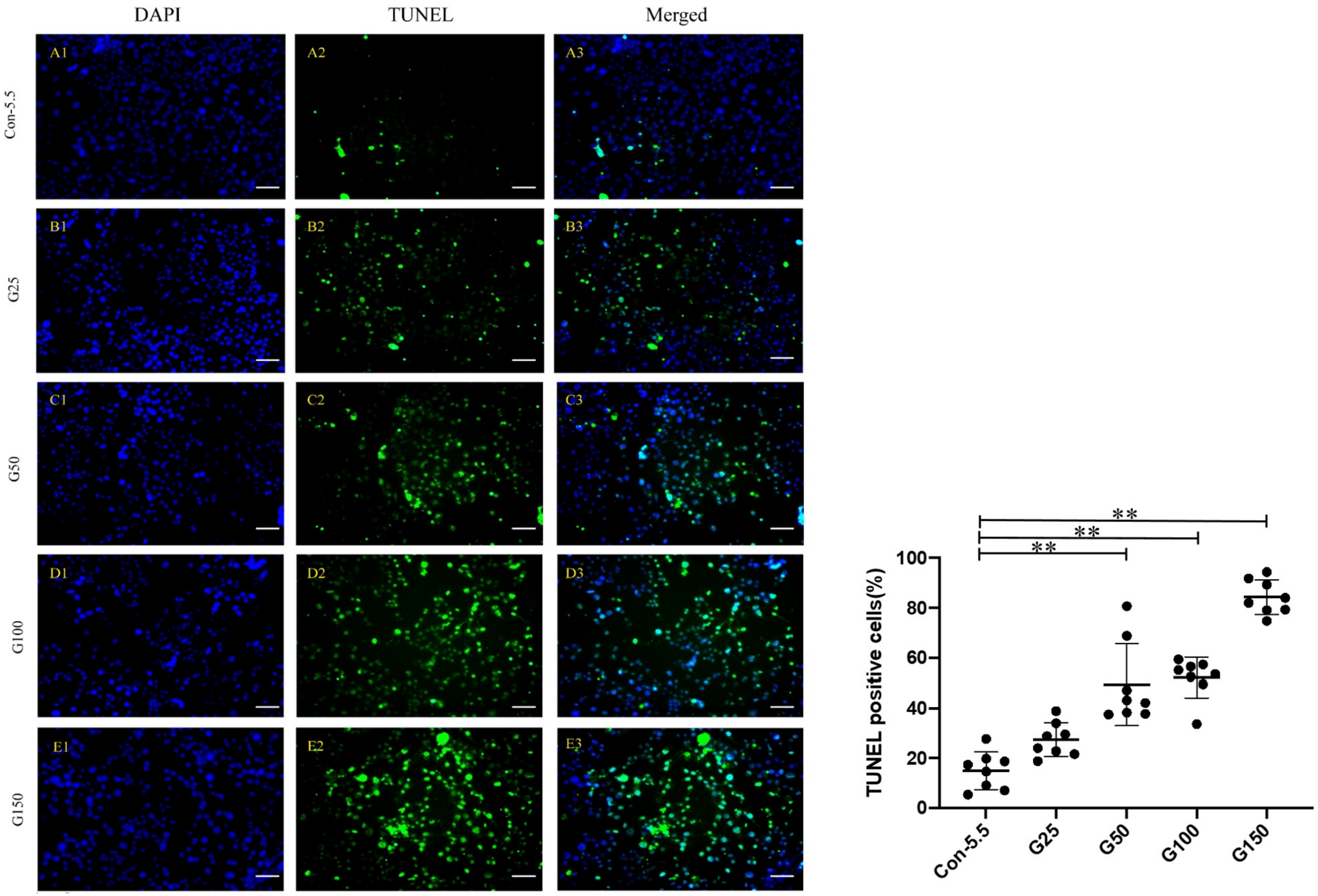

Supplemental Figure 7. Representative fluorescence images and quantification of TUNEL-positive cells. TUNEL staining(green, A2-E2), DAPI staining(blue, A1-E1), and merged image of RGCs with different treatments. Merged images were presented as A3-E3. Con-5.5, G25- G150: concentration of glucose (5.5, 25, 50, 100 and 150 mmol/l, respectively).Scale bar: 200 μm(A). *$P < 0.05$; **$P < 0.01$.
